# Assessment of the Influence of Technology-Based Distracted Driving on Drivers’ Infractions and Their Subsequent Impact on Traffic Accidents Severity

**DOI:** 10.3390/ijerph18137155

**Published:** 2021-07-04

**Authors:** Susana García-Herrero, Juan Diego Febres, Wafa Boulagouas, José Manuel Gutiérrez, Miguel Ángel Mariscal Saldaña

**Affiliations:** 1 Escuela Politécnica Superior, Universidad de Burgos, 09006 Burgos, Spain; wafa.boulagouas@umc.edu.dz (W.B.); mariscal@ubu.es (M.Á.M.S.); 2 Department of Chemistry and Exact Sciences, Universidad Técnica Particular de Loja, 110107 Loja, Ecuador; jdfebres@utpl.edu.ec; 3 Consejo Superior de Investigaciones Científicas, 39005 Santander, Spain; manuel.gutierrez@unican.es

**Keywords:** road traffic accidents, technology-based distractions, aberrant infractions, speed infractions, bayesian network, traffic accidents severity

## Abstract

Multitasking while driving negatively affects driving performance and threatens people’s lives every day. Moreover, technology-based distractions are among the top driving distractions that are proven to divert the driver’s attention away from the road and compromise their safety. This study employs recent data on road traffic accidents that occurred in Spain and uses a machine-learning algorithm to analyze, in the first place, the influence of technology-based distracted driving on drivers’ infractions considering the gender and age of the drivers and the zone and the type of vehicle. It assesses, in the second place, the impact of drivers’ infractions on the severity of traffic accidents. Findings show that (i) technology-based distractions are likely to increase the probability of committing aberrant infractions and speed infractions; (ii) technology-based distracted young drivers are more likely to speed and commit aberrant infractions; (iii) distracted motorcycles and squad riders are found more likely to speed; (iv) the probability of committing infractions by distracted drivers increases on streets and highways; and, finally, (v) drivers’ infractions lead to serious injuries.

## 1. Introduction

The road transportation system presents a high-risk system that threatens people’s lives every day [1]. The severity of traffic accidents raises many social direct and indirect problems, physical and mental health disorders, economic expenses, and massive damage to the surroundings and properties [2]. The World Health Organization estimates that approximately 1.35 million people die in road traffic accidents each year (on average, 3700 people lose their lives per day), and 20 to 50 million more people suffer non-fatal injuries, which often lead to long-term disabilities [3]. In this regard, studies on the features of road safety concluded that traffic accidents occur mainly due to human factors, road infrastructure, environmental aspects, and their interactions [4,5]. Nevertheless, a growing body of research suggests that human factors, e.g., speeding, drink-driving, distracted driving, have the strongest influence and are responsible for 80–90% of road traffic accidents [6,7,8,9]. Indeed, past research reported that distracted driving contributes to over half of inattention traffic accidents [10]. This is supported by an explosion of studies. For instance, in the USA, the National Highway Safety Administration estimated that distracted driving is responsible for approximately 10% of all fatal traffic accidents [11]. Similarly, in Spain, the General Traffic Department, reported that distracted driving contributed to approximately 28% of police-reported fatal traffic accidents [12].

Placing greater importance on the fact that secondary tasks that compete for the driver’s attention and potentially degrade their perception and their ability to interpret the information they receive continually from a changing roadway [13,14], in the recent past, many researchers and traffic transportation experts have deeply studied aspects of distracted driving and concluded that a majority of distracted driving is related to new technologies. Furthermore, a review of the current literature on distracted driving found a specific focus on mobile phone-related distractions. For instance, a survey study reported that 42% of drivers confirmed that they answer their mobile phones when driving, and 56% admitted to continuing to drive while completing the conversation [15]. An observational study of 6578 drivers on randomly selected urban roads in Spain found that 20% of the observed drivers engaged in secondary tasks including talking on handheld mobile phones (i.e., 1.3%) [16].

A simulator study investigating the relationship between performing a secondary task (e.g., mobile phone use) and driving performance reported that engaging in a secondary task influences longitudinal and lateral control of the vehicle and leads to higher speeds [17]. Moreover, an examination of the chance of drivers colliding increases twelvefold when they handled their mobile phones [18]. Furthermore, it has been found that the effects of the use of mobile phones on drivers’ reactions are worse than driving under a 0.08% blood alcohol level [19].

Another experimental study analyzed the effects of the use of mobile phones on the reaction time of drivers in dangerous situations (pedestrian crossing and road crossing by parked vehicles, in particular) found that the use of mobile phones leads up to 204% increments in reaction times, proving that distractions decrease the driving performance [20].

However, an estimation of the contribution of distracted driving in traffic accidents causality and its impact on other unsafe driving behaviors is complex. Indeed, there are the following three major challenges [21,22]: (i) obtaining reliable data about pre-accident conditions is difficult, (ii) there is a lack of systemic reporting, and (iii) there are inconsistencies in the definitions, classifications, and approaches used. Dealing with these challenges, many scholars have reviewed traffic accident assessment tools and advanced techniques and algorithms to increase the efficiency and effectiveness of road safety protective measures [23,24,25]. Recently, many researchers have moved toward correlating the analysis methods with prediction techniques to model interactions between risk factors and predict potential impacts on causalities, frequencies, and the severity of traffic accidents. Such a combination considers several parameters to analyze the current conditions that are, therefore, assessed using the mean of the prediction models that contribute to mitigate the magnitude of traffic accidents and enhance the transportation system and safety strategies [26]. Among the emergent techniques, there are the Grey System Theory and Markov Model [2], Data Mining Techniques [27], Structural Time Series [28], Logistic Regression Analysis [29], and Bayesian Networks [30]. 

Building on these attempts, this paper focuses particularly on analyzing the influence of technology-based distracted driving on drivers’ infractions and assesses their subsequent impact on the severity of traffic accidents employing recent data on road traffic accidents in Spain. 

The present study is designed to provide relevant pieces of evidence on the relationship between technology-based distracted driving and other infractions. The assessment is therefore extended to evaluate the relationships between the infractions of the distracted drivers and the severity of traffic accidents and investigate the impact of a set of factors grouped into demographics, type of vehicle, and zone. 

An assessment of the influence of technology-based distractions on drivers’ infractions allows the accident risk to be estimated. In other words, it allows for, first, an appreciation of the proportion by which the probability of committing aberrant infractions and speeding would be expected to increase, provided that the driver is distracted. Second, it captures the infractions that lead to serious traffic accidents resulting in fatalities, while incorporating relevant parameters. 

The remainder of this paper is arranged as follows: Section 2 reviews distracted driving; Section 3 sums up the data and methodology of the study; Section 4 provides the results of the study; Section 5 discusses the results and puts forward main findings, limitations, and future research guideline, while Section 6 concludes the paper. 

## 2. Background

Distracted driving has emerged as a major phenomenon that compromises traffic safety. It adversely impacts driving performance, increases reaction time, and reduces control over the vehicle; thus, it accounts for 25% of severe motor vehicle accidents, leading to significant morbidity and mortality [31]. In terms of definition, distracted driving refers to the inattention of drivers and their focus on other competing activities while operating a motor vehicle, for instance, talking, smoking, texting, putting on make-up, reading, eating, using mobile phones, etc. [32].

In light of the past research, distracted driving could be either intentional and voluntary, which occurs when drivers divert their attention from the driving tasks, or involuntary due to a failure to ignore non-related stimuli that motivates drivers to become distracted [33,34,35].

Distractors have been grouped into the following four main categories [36,37]: (i) visual, which implies taking the eyes off the road; (ii) auditory, which prevents making the best use of hearing; (iii) manual, which considers taking the hands off the wheel; and, finally, (iv) cognitive, when losing concentration on driving.

Moreover, distractions have been classified into [38,39,40] (i) in-vehicle distractions, such as using mobile phones, interacting with an entertainment system, an iPod, radio, DVD player, operating navigation system, etc., and (ii) on-road distractions, such as roadside advertisements, crash scenes, digital billboards, etc.

Although other potential distractions are still of interest, technology-based distractions have attracted the focus of researchers who thoroughly investigated the risks associated with the use of technology devices (e.g., smartphones, wearable devices, portable devices, and in-vehicle information systems) while driving and used many approaches for risk estimation, for instance [41], surveys, simulators, phone records, on-road testing, and naturalistic studies. 

A study analyzing the contribution of distracted driving to traffic accidents in the United States reported that 16% of motor vehicle crashes in which people were killed and 20% of those that caused injuries were a result of distracted driving [13]. Furthermore, a synthesis of previous studies on mobile phone use and its effects on traffic safety has identified mobile phones as leading sources of distraction that reduce driving performance as they decrease the reaction time of the drivers [17,42,43,44]. After investigating the driving performance of distracted drivers, many studies have concluded that distracted drivers are more likely to engage in unsafe driving behaviors and several errors and violations increase, for instance, speeding violations, right-of-way violations, and failure to stop at stop signs and red lights [45,46,47]. For a better understanding of the characteristics of distracted drivers and the frequency of being engaged in distractions, many researchers have investigated the demographics of distracted drivers. Indeed, in terms of gender, a study conducted in the UK reported that no differences have been found for many types of distractions (e.g., mobile phone conversations) [48]. Furthermore, the results of this study considered the age of the driver as a crucial predictor for most of the studied distractions (including technology-based distractions), concluding that older drivers are less likely to be distracted, unlike younger drivers. Similarly, a roadside observational survey in Melbourne [49] has found that gender does not influence distracted driving, in contrast to age, where it has been suggested that young or middle-aged drivers are the predominant group in distracted driving.

Second, despite the increasing body of research on the effects of distracted driving on traffic accidents, a large part of the literature relies mostly on survey, observational, and simulator studies, which suffer a set of limitations. First, survey studies, most of the time, target one particular group of drivers (e.g., young, male, female, etc.) or analyze distracted driving by accumulating all the groups together. Furthermore, the data collected in such survey and/or interview studies are likely to be biased [50]. Even though observational studies are typically conducted at particular places, allowing sufficient time for the observer to capture the behaviors of the drivers, they, in turn, limit the data to one location along the roadway [51]. Finally, driving simulator experiments are safe assessment procedures and provide a more realistic environment; however, traffic situations are unpredictable and uncontrollable and, therefore, simulators have limited fidelity [52].

The present study is designed to adequately remove, first, the bias of underreporting and after that deploys a machine learning technique to adequately investigate the influence of distracted driving on drivers’ infractions considering the drivers’ characteristics, the type of vehicle, and the zone, and assess the impact on the severity of traffic accidents.

## 3. Materials and Methods

### 3.1. Data Description

For this study, a dataset has been prepared using official data of traffic accidents that occurred in Spain in a period of four years (2016–2019) provided by the Spanish National Transportation Department. The data have been gathered by the Civil Guard General Directorate or local police officers and include information related to the traffic accident, for instance, the date, location, type of vehicles involved in the traffic accidents, demographics of the drivers, number of fatalities, trip purpose, violations, and errors, etc.

The current sample of this analysis includes 410,974 traffic accidents involving 666,504 drivers. Details of the study sample are given in Table 1.

The dataset contains information about the severity of traffic accidents presented in (Table 2). This information is aggregated into the following two groups: (i) Fatal accidents (FA) resulting in serious injuries (SI) or fatalities to the drivers involved in serious accidents (55545), and (ii) Minor accidents (MA) causing no injuries (NI) or resulting in slight injuries to the drivers involved in these minor accidents. 

The collected information from the dataset about technology-based distractions and drivers’ infractions is given in Table 3. 

### 3.2. Bias Identification

The dataset used to conduct this study was gathered by the Civil Guard General Directorate and local police officers who register information on traffic accidents, which is always incomplete. Indeed, many researchers have confirmed that as data on traffic accidents involving distracted drivers are collected from traffic accident reports, the real influence of this later on driving behaviors goes underestimated [53,54]. Furthermore, distractions are hard to prove using statistics from the police [55], because, in general, police officers only look for distractions when the consequences of traffic accidents are serious and, therefore, many distracted driving cases may not be recorded. Thus, scientific studies lead to unreliable conclusions. 

To address the reporting biases, a methodology proposed in recent research [46] has been adopted. It is based on the introduction of a “dummy variable” into the model to isolate homogeneous subsamples and generate valid model and unbiased parameter estimations. Accordingly, the frequencies of “technology-based distractions” have been thoroughly analyzed. Particularly, differences between the percentage of drivers involved in severe accidents knowing the states (i.e., being or not distracted) and the percentage of drivers involved in severe accidents having unknown states (i.e., unspecified) are computed (10.86% versus 7.40%, respectively) (Table 4). The differences are found to be significant; therefore, the variable “technology-based distractions” is biased, and a dummy variable is introduced to differentiate homogeneous cases. In the rest of the paper, the sensitivity analysis results will only show the probabilities of drivers’ infractions in traffic accident severity in a case of the state, “presence or absence of technology-based distractions” of dummy variable technology-based distractions. In this way, the subsamples containing unknown cases about the technology-based distractions are eliminated. 

### 3.3. Bayesian Networks

In the present study, Bayesian Networks have been deployed to model the influence of distracted driving on the severity of traffic accidents and unsafe driving behaviors. Over the last few decades, the Bayesian approach has been extensively applied to traffic safety studies, for instance, an assessment of road safety [56,57], an assessment of the influence of seatbelt use on the severity of traffic accidents [58], an estimation of committing an infraction due to mobile use [59], music distraction among young drivers [60], a prediction of traffic accidents [61], etc. 

The Bayesian Networks model is a graphical inference methodology capable of predicting the future behavior of a particular variable based on past experience learned from the historical data source. Furthermore, they consist mainly of the following:

- The qualitative aspect of the Bayesian Networks given by a directed acyclic network generally denoted as DAG (V, E), consisting of nodes (V) representing the variables related with directed edges (E), denoting the dependencies between the variables;
- The quantitative aspect consists of the conditional probability of each node, where every node has parents and has a conditional probability table expressing the dependencies of the father nodes. Therefore, the joint probability distribution is expressed as follows:
  (1) $$P\left(X_{1},\ldots ,\;X_{n}\right)=\overset{n}{\underset{i=1}{\prod }}P\left(X_{1}|X_{i-1},\ldots ,X_{n}\right)$$
  (2) $$P\left(X\right)=\overset{n}{\underset{i=1}{\prod }}P(X_{i}|pa_{i})\;$$
  where i=1,2,…, n, $X=\left\{X_{1},\;X_{2},\;\ldots ,X_{n}\right\}$ is a set of variables of the Bayesian Network, P is a set of local probability distributions associated with each variable, $X_{i}$ refers to the variable node and, $pa_{i}$ is the father node of $X_{i}$.

Many types of software could be used to build the Bayesian network, among them we cite the following [62]: BayesBuilder, JavaBayes, and Bayes Net Toolbox, which were used in the present study.

### 3.4. Network Validation

To measure the performance of the obtained Bayesian Network, a ten-fold cross-validation has been conducted. K-fold cross-validation is a powerful means of testing the success rate, accuracy, and robustness of models used for classification [63]. It consists of randomly partitioning the dataset into ten subsets of equal size; then each subset, in turn, is used to validate the model fitted on the remaining k-1 subsets. The evaluation of the obtained models is performed using the Area Under the ROC (Receiver Operating Characteristic) Curve—named AUC—that plots the following two parameters: True Positive Rate and False Positive Rate that yield the performance of a classification model. The standard scores range from 0 (opposite and wrong prediction) to 0.5 (random prediction) and 1 (perfect prediction).

## 4. Results

### 4.1. Validation of Bayesian Network

**Figure 1:**
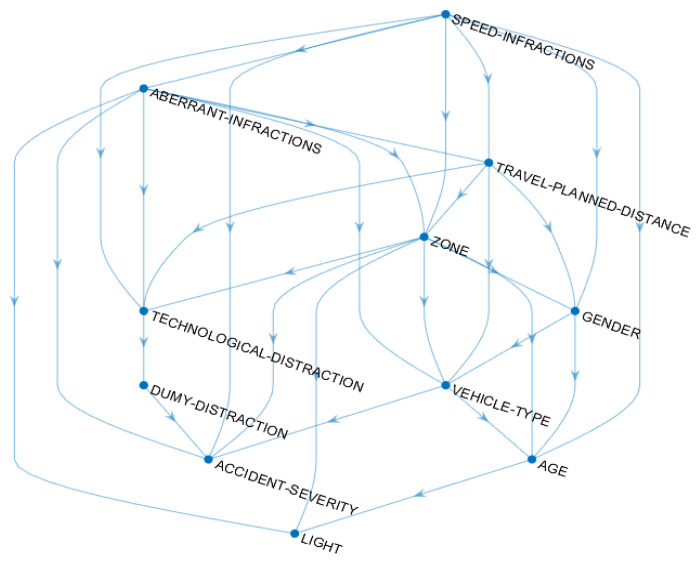
Obtained directed acyclic graph corresponding to the study variables.

The results of the performance evaluation of the learned Bayesian Network (Figure 1) are given in Table 5.

The AUC score metrics obtained a range from 0.62 to 0.99 for the variables, which confirms the high robustness of the Bayesian Network.

The directed acyclic graph of Figure 1 specifies the joint distribution and represents the dependences/independences between the study variables. Indeed, in the lower right part of the graph, the largest number of study variables such as the zone, gender, vehicle type, and age can be observed in a grouped way. These variables have direct relationships with the infractions’ variables before relating, directly or indirectly, to the severity of the accident. At the top of the graph, the infractions’ variables are directly related to the technology-based distraction variable. Furthermore, aberrant and speed infractions are related to the dummy variable, which is directly related to the severity of the traffic accidents.

### 4.2. Sensitivity Analysis

#### 4.2.1. Assessment of the Influence of Technology-Based Distractions on Drivers’ Infractions

Estimation of the “a priori” probabilities of drivers’ infractions considering the technology-based distractions is given by the sensitivity analysis results in Table 6.

The results in Table 6 show that the probability of committing aberrant infractions increases significantly (from 23.57 to 66.75%) given the fact that the drivers are distracted. Interestingly, these results show that the probability of not committing aberrant infractions increased notably (from 33.25 to 76.43%) when the drivers are not distracted.

Similarly, the sensitivity analysis results in Table 6 show that drivers are more likely to speed when they are distracted. Indeed, the probability increases from 7.78 to 14.45%. Moreover, the probability of respecting the speed limit increases from 85.55% to 92.22% when drivers are not distracted.

The results also show that technology-based distractions are more likely to affect aberrant infractions than speed infractions.

#### 4.2.2. Assessment of the Influence of Technology-Based Distractions on Drivers’ Infractions Considering the Age and Gender of the Drivers

The influence of distracted driving on drivers’ infractions considering the effect of demographics is given by the sensitivity analysis results in Table 7.

According to these results, the probability of not committing aberrant infractions, considering the fact that the drivers are not distracted, increases for drivers younger than 60 years old. Similarly, it has been found that the probability of not speeding, considering the fact that the drivers are not distracted, increases for drivers older than 40 years old and more significantly in the case of elderly drivers (from 92.22 to 95.39%).

With regard to the gender, the results in Table 8 show that the probability of not speeding, considering the fact that the drivers are not distracted, increases notably in the case of females (from 92.22 to 94.08%).

In terms of aberrant infractions, the sensitivity analysis results show that the probabilities increase given the fact that the drivers are distracted (from 66.75 to 68%) in the case of younger drivers (<25 years old). Similarly, it has been found that the probability of speeding for distracted drivers increases, interestingly, in the case of younger drivers (<25 years old) (from 14.45 to 27.14%) and decreases significantly in the case of older drivers (from 14.45 to 8.28%).

#### 4.2.3. Assessment of the Influence of Technology-Based Distractions on Drivers’ Infractions Considering the Zone and Type of the Vehicle

The results of the estimation of the influence of technology-based distractions on drivers’ infractions considering the zone and type of the vehicle are given in Table 8. The results show that the probability that drivers do not commit aberrant infractions increases in the case of motorcycles, quads, and quadricycles (from 76.43 to 86.58%) and other types of vehicles (from 76.43 to 85.48%), considering the fact that the drivers are not distracted.

In contrast, the results show that the probability of committing aberrant infractions increases more significantly in the case of distracted drivers of cars, vans, and all-terrain vehicles (from 66.75 to 68.91%).

With regard to speed, similarly, the results of the sensitivity analysis show that the probability of respecting the speed limits increases significantly in the case of non-distracted motorcycle riders and drivers of other vehicles (from 92.22 to 93.46%, and from 92.22 to 95.67%, respectively). Moreover, the probability of speeding increases, logically, in the case of distracted motorcycle riders.

The results of the effect of the zone on the behaviors of distracted drivers confirm that distracted drivers commit aberrant infractions on highways and streets (the probabilities increase from 66.75 to 79.80% and from 66.75 to 81.11%, respectively). Nevertheless, distracted drivers are more likely to not respect the speed limit on highways (the probability increases significantly from 14.45 to 39.59%).

#### 4.2.4. Assessment of the Influence of Drivers’ Infractions on Traffic Accident Severity

The severity of traffic accidents due to aberrant infractions or speeding is not always reported in the case of minor traffic accidents. In other words, in such accidents, the police officers are not used to thoroughly fill in the traffic accident report and, most of the time, they do not provide details on whether the drivers involved in minor accidents had committed aberrant infractions or speed infractions.

The influence of drivers’ infractions on the severity of traffic accidents is given by the sensitivity analysis results in Table 9.

The results in Table 9 show that the probability that the severity of injuries is serious, resulting in fatalities, increases, given the fact that the drivers committed aberrant infractions (from 9.38 to 9.69%) and more significantly in the case of speeding (the probability increases from 9.03 to 17.89%).

## 5. Discussions

Traffic accidents have become a major health public issue and preservation of the lives of drivers, passengers and users is among the main concerns of communities around the world. The literature revealed the fact that numerous factors contribute to and influence the occurrence of these accidents and the severity of injuries [64,65,66]. Although the continuous efforts of governments and numerous traffic safety policies being issued to control traffic accidents, the rates of disabilities, fatalities, and injuries continue to increase dramatically. Therefore, it has been extensively recommended to analyze the different risk factors influencing traffic accident severity to yield more patterns and polish knowledge to effectively and efficiently prevent road accidents and ameliorate traffic safety.

In this study, we focused, particularly, on the assessment of the influence of technology-based distractions on the performance of drivers behind the wheel. The contribution of this research is twofold. First, it deployed machine learning technique algorithms that, taking advantage of advancements in information technology, allow traffic accidents to be predicted and detect the role of different risk factors in traffic accident scenarios. Second, it overviewed the relationships between the technology-based distractions and the infractions of drivers with full consideration of the impact of gender, age, zone, and the type of vehicle.

Given the crucial aspect of traffic safety, i.e., the prediction of the most important risk factors impacting the occurrence of traffic accidents and influencing the severity of the outcomes, in the present study, a predictive model has been developed to assess the influence of technology-based distractions on the traffic accidents’ severity and unsafe driving behaviors considering the effect of a set of selected risk factors using Bayesian Networks methodology. Indeed, the obtained Bayesian model presented the knowledge in the form of joint probabilities distributions of the study variables, which is vital to make effective and efficient decisions that avoid the drivers’ infractions resulting from technology-based distractions and reduce the severity of injuries to the drivers and passengers.

To conduct this investigation, a recent database over four years (from 2016–2019) on traffic accidents that occurred in Spain was used. According to the sensitivity analyses, the findings indicated that technology-based distracted driving has a significant effect on both the aberrant infractions and speeding. Indeed, the study found that the presence of distractions notably increases the probability of committing aberrant infractions and speeding. This is in line with the literature [67,68] that overviewed the characteristics of distracted driving, which has agreed that new technologies can absorb drivers’ attention and reduce their abilities to judge driving demands and disrespect driving safety requirements that lead, most of the time, to aberrant infractions and unsafe behaviors.

With regard to the risk factors related to the demographics of the drivers involved in traffic accidents, vehicle, and zone, the findings of the present study reported increased probabilities as regards the infractions of distracted young drivers (under 25 years old). These findings coincide with many conclusions of existing studies [69,70,71], which had reported similar observations on risky driving behaviors and violations of young drivers who are more susceptible to be involved in fatal crashes due to distraction activities (e.g., mobile phone use, radio, DVD, etc.). In this context, young drivers are inexperienced and easily distracted by interactions with music devices, texting and conversations on mobile phones resulting in slower reaction times [72].

The sensitivity analysis results of the present study also showed that there are no significant differences in the probabilities of the impact of the gender of drivers on the driving behaviors of distracted drivers.

Furthermore, it has been found that speeding will lead to severe traffic accidents, leading to serious injuries and/or fatalities. These results are tightly associated with the findings of many previous researchers [73,74] that confirmed that speed increases the chance of traffic accidents that result in greater severity.

With regard to the influence of the zone and type of the vehicle factors, the sensitivity analysis showed that distracted drivers are more likely to speed on highways, whereas on streets, distracted drivers are more susceptible to commit aberrant infractions. Moreover, it has been found that the probability of motorcycle riders speeding increases because they are distracted, while car drivers are more likely to commit aberrant infractions. Similar findings reported that mortalities on highways and outside the urban center are mainly due to speed-related crashes [75,76]. Furthermore, an investigation into drivers’ violations found that speeding infractions represent 80% of registered traffic violations on highways [77].

For future research, it is recommended to extend the current study by considering other risk factors. Such consideration would give wider context and more shreds of evidence on the influence of distracted driving that would help improve and enhance traffic safety more effectively and efficiently.

## 6. Conclusions

Distracted driving is a growing threat to road safety and accounts for a significant number of serious injuries and deaths in traffic accidents. In this paper, the distracting effects of various technologies on driving performance have been assessed using Bayesian Networks. Unlike other studies, the assessment was then extended to analyze the influence of the driver’s infractions on the severity of traffic accidents. The design of this study considered the driver’s characteristics, the type of vehicle, and zone.

The results of this study documented a strong link between technology-based distracted driving and aberrant and speed infractions. Moreover, the findings showed that these infractions have a direct impact on the severity of traffic accidents. Furthermore, this study specifically found that young drivers are more likely to be distracted.

The deployment of Bayesian Networks yielded a representative graphical structure of the relationships among the technology-based distractions, drivers’ infractions, and traffic accidents’ severity. It contributed to overcoming the observed limitations of most research that has used regression models. Thus, machine learning techniques proved to be more suitable for prediction models in traffic safety problems.

## Figures and Tables

**Table 1:** Frequencies of the study sample.

	Variables	Number of Cases				
**Traffic Accident**	**Vehicle Type**	**2016**	**2017**	**2018**	**2019**	**Total**
	Car, van, all-terrain vehicles	120,831	120,261	119,755	118,491	479,338
	Motorcycles, quads, quadricycles	35,222	36,051	36,319	37,467	145,059
	Heavy vehicles	8906	8901	9176	8873	35,856
	Other vehicles	922	759	1039	3531	6251
	Total	165,881	165,972	166,289	168,362	666,504
**Drivers** **Demographics**	**Age**					
	Y < 25	21,983	21,350	20,707	21,432	85,472
	25 ≤ Y ≤ 40	63,188	61,476	61,191	60,538	246,393
	40 ≤ Y ≤ 60	59,857	61,660	63,696	65,369	250,582
	Y > 60	17,399	18,102	18,333	19,010	72,844
	Unspecified	3454	3384	2362	2013	11,213
	Total	165,881	165,972	166,289	168,362	666,504
	**Gender**					
	Male	119,878	120,447	120,920	122,407	483,652
	Female	445,44	44,225	44,772	45,407	178,948
	Unspecified	1459	1300	597	548	3904
	Total	165,881	165,972	166,289	168,362	666,504

**Table 2:** Traffic accident severity distribution.

**Traffic** **Accident** **Severity**	**Severity Level**	**Number of Cases**				**Total**
		**2016**	**2017**	**2018**	**2019**	
	M/NI	151,446	151,595	152,757	155,161	610,959
	SI/F	14,435	14,377	13,532	13,201	55,545
	Total	165,881	165,972	166,289	168,362	666,504

SI/F: Serious Injuries and/or Fatalities M/NI: Minor and/or No Injuries.

**Table 3:** Frequencies of technology-based distractions and drivers’ infractions.

**Infractions**	**Drivers’ Infractions**	**Number of Cases**				
		**2016**	**2017**	**2018**	**2019**	**Total**
	**Aberrant infractions**					
	No infractions	54,405	52,131	52,054	62,566	221,156
	Aberrant infractions	34,558	35,623	36,260	40,083	146,524
	Unspecified	76,918	78,218	77,975	65,713	298,824
	Total	165,881	165,972	166,289	168,362	666,504
	**Speed infractions**					
	No speed infractions	70,573	69,451	67,252	79,677	286,953
	Speed infractions	8957	8154	8395	8117	33,623
	Unspecified	86,351	88,367	90,642	80,568	345,928
	Total	165,881	165,972	166,289	168,362	666,504
**Distracted Driving**	**Technology-based** **distractions**					
	No distractions	41,766	41,790	41,944	42,634	168,134
	Technology-based distractions	881	1029	1024	1114	4048
	No technology-based distractions or unspecified	123,234	123,153	123,321	124,614	494,322
	Total	165,881	165,972	166,289	168,362	666,504

**Table 4:** Dummy Variable Frequencies.

**Dummy Variable Technology-Based Distractions**	**States**	**Number of Cases**				**Total**	**Percentage**	**SI/F**
		**2016**	**2017**	**2018**	**2019**			
	Presence or absence of technology-based distractions	42,647	42,819	42,968	43,748	172,182	25.83%	10.86%
	Unknown	123,234	123,153	123,321	124,614	494,322	74.17%	7.40%
	Total	165,881	165,972	166,289	168,362	666,504	/	

**Table 5:** Validation results of the 10-fold cross-validation.

Variables	Accident Severity		Aberrant Infractions		Speed Infractions		Technology-Based Distraction	
**States**	SI/F	M/NI	No	Yes	No	Yes	No	Yes
**AUC scores**	0.62	0.62	0.88	0.77	0.90	0.77	0.99	0.93

**Table 6:** Sensitivity analysis for the influence of technology-based distractions on drivers’ infractions.

Technology-Based Distractions	Aberrant Infractions		Speed Infractions	
States	No	Yes	No	Yes
No	76.43%	23.57%	92.22%	7.78%
Yes	33.25%	66.75%	85.55%	14.45%

**Table 7:** Sensitivity analysis for the influence of technology-based distractions on drivers’ infractions considering the influence of demographics.

**Demographics**	**Technology-Based Distractions**	**Aberrant Infractions**		**Speed Infractions**	
**Age**	**States**	**No**	**Yes**	**No**	**Yes**
Y < 25	No	76.65%	23.35%	85.66%	14.34%
	Yes	32.00%	68.00%	72.86%	27.14%
25 ≤ Y ≤ 40	No	76.50%	23.50%	91.39%	8.61%
	Yes	33.18%	66.82%	84.10%	15.90%
40 ≤ Y ≤ 60	No	76.61%	23.39%	93.96%	6.04%
	Yes	33.82%	66.18%	88.94%	11.06%
Y > 60	No	75.54%	24.46%	95.39%	4.61%
	Yes	33.66%	66.34%	91.72%	8.28%
**Gender**	**Technology-Based Distractions**	**Aberrant Infractions**		**Speed Infractions**	
	**States**	**No**	**Yes**	**No**	**Yes**
Male	No	76.38%	23.62%	91.45%	8.55%
	Yes	33.21%	66.79%	84.25%	15.75%
Female	No	76.56%	23.44%	94.08%	5.92%
	Yes	33.45%	66.55%	88.91%	11.09%

**Table 8:** Sensitivity analysis for the influence of technology-based distractions on drivers’ infractions considering the influence of the zone and vehicle type.

**Variables**	**Technology-Based Distractions**	**Aberrant Infractions**		**Speed Infraction**	
**Vehicle Type**	**States**	**No**	**Yes**	**No**	**Yes**
Car/Van/All Terrain	No	73.73%	26.27%	92.02%	7.98%
	Yes	31.09%	68.91%	85.80%	14.20%
Motorcycles/quads/quadricycles	No	86.58%	13.42%	93.46%	6.54%
	Yes	43.83%	56.17%	84.56%	15.44%
Heavy vehicles	No	78.28%	21.72%	90.44%	9.56%
	Yes	39.70%	60.30%	84.73%	15.27%
Other vehicles	No	85.48%	14.52%	95.67%	4.33%
	Yes	34.90%	65.10%	85.37%	14.63%
**Zone**	**Technology-Based Distractions**	**Aberrant Infractions**		**Speed Infraction**	
	**States**	**No**	**Yes**	**No**	**Yes**
Road	No	76.19%	23.81%	89.03%	10.97%
	Yes	41.06%	58.94%	85.64%	14.36%
Street or similar	No	76.74%	23.26%	96.97%	3.03%
	Yes	18.89%	81.11%	85.50%	14.50%
Highway	No	83.82%	16.18%	94.19%	5.81%
	Yes	20.20%	79.80%	60.41%	39.59%

**Table 9:** Sensitivity analysis for the influence of the drivers’ infractions on the severity of traffic accidents.

Drivers’ Infractions	Severity of Traffic Accidents	
**Aberrant infractions**	*M/NI*	*SI/F*
No	90.62%	9.38%
Yes	90.31%	9.69%
**Speed infractions**		
No	90.97%	9.03%
Yes	82.11%	17.89%
